# An overview of RNA splicing and functioning of splicing factors in land plant chloroplasts

**DOI:** 10.1080/15476286.2022.2096801

**Published:** 2022-07-11

**Authors:** Xuemei Wang, Jingyi Wang, Simin Li, Congming Lu, Na Sui

**Affiliations:** a Shandong Provincial Key Laboratory of Plant Stress, College of Life Sciences, Shandong Normal University, Jinan, Western Shandong, China; b State Key Laboratory of Crop Biology, College of Life Sciences, Shandong Agricultural University, Taian, Western Shandong, China

**Keywords:** Chloroplast, intron, RNA splicing, RNP, splicing factor

## Abstract

RNA splicing refers to a process by which introns of a pre-mRNA are excised and the exons at both ends are joined together. Chloroplast introns are inherently self-splicing ribozymes, but over time, they have lost self-splicing ability due to the degeneration of intronic elements. Thus, the splicing of chloroplast introns relies heavily on nuclear-encoded splicing factors, which belong to diverse protein families. Different splicing factors and their shared intron targets are supposed to form ribonucleoprotein particles (RNPs) to facilitate intron splicing. As characterized in a previous review, around 14 chloroplast intron splicing factors were identified until 2010. However, only a few genetic and biochemical evidence has shown that these splicing factors are required for the splicing of one or several introns. The roles of splicing factors are generally believed to facilitate intron folding; however, the precise role of each protein in RNA splicing remains ambiguous. This may be because the precise binding site of most of these splicing factors remains unexplored. In the last decade, several new splicing factors have been identified. Also, several splicing factors were found to bind to specific sequences within introns, which enhanced the understanding of splicing factors. Here, we summarize recent progress on the splicing factors in land plant chloroplasts and discuss their possible roles in chloroplast RNA splicing based on previous studies.

## Introduction

**Figure 1.**
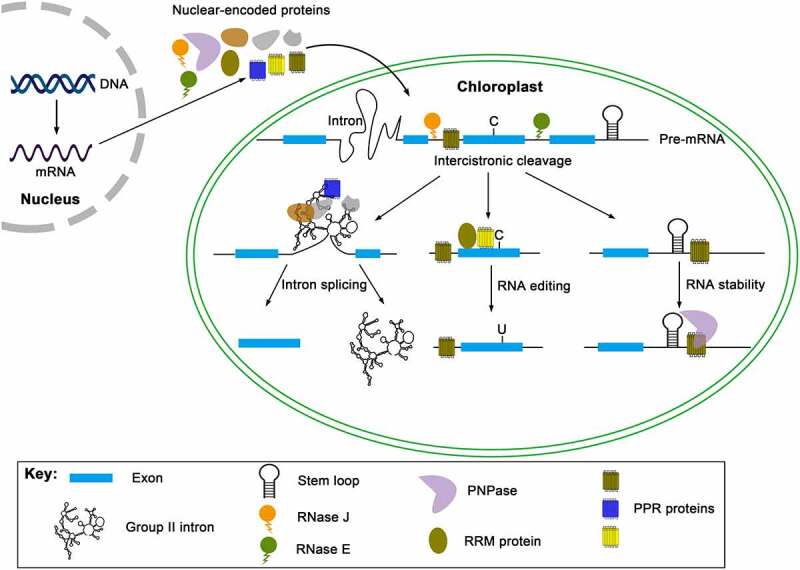
Post-transcriptional control of chloroplast gene expression in land plant chloroplasts. The post-transcriptional processing of chloroplast RNAs includes RNA cleavage of precursor mRNA (pre-mRNA), intron splicing, RNA editing, and RNA stability. Various nuclear-encoded proteins are implicated in these processes. RNase J and RNase E may act as endonucleases to cleave intercistronically. One or more sequence-specific RNA-binding proteins bind to intron RNA, and other splicing factors are recruited to intron RNAs, and the binding of these splicing factors assists the intron folding and splicing. The C-to-U editing is catalysed by PPR and RIP/MORF proteins, and a specific PPR protein targets an editing site by the cis sequence upstream of the editing site. The binding of RNA binding proteins can also protect RNA from exonucleolytic cleavage.

Land plant chloroplasts evolved from endosymbiotic cyanobacteria, and chloroplast genome has retained some prokaryotic properties while also evolved some eukaryotic properties. The features of chloroplast genome have determined the complexity of the chloroplast gene expression regulation. The inconsistence of chloroplast gene transcription rates and steady-state levels of mature transcripts indicates that the post-transcriptional control is a critical step in regulating chloroplast gene expression [1–3]. The post-transcriptional processes include pre-mRNA cleavage, RNA splicing, RNA editing, and RNA stability [1] (see Fig. 1). Many nuclear-encoded proteins regulate these processes, which is essential to converting pre-mRNAs into mature mRNAs. In this review, we have focused on chloroplast intron splicing.

The chloroplast genome underwent intron gain during the evolution from cyanobacteria to land plants [4,5]. Based on the distinct structures and splicing mechanisms of chloroplast introns, these introns were categorized into group I and group II introns [6–8]. The presence of introns in some chloroplast genes that are essential for photosynthesis and chloroplast gene expression makes efficient RNA splicing necessary.

Group II introns, as mobile genetic elements, contain a catalytic RNA and an intron-encoded protein (IEP), the intron RNA catalyses RNA splicing reactions, which are similar to those of the spliceosomal introns, on the other hand, the role of IEP with reverse transcriptase activity is to assist RNA splicing by stabilizing the catalytically active RNA structure (known as maturase activity) and function in intron mobility [9–11]. However, group II introns in plant chloroplasts underwent degenerations in RNA structures and loss or degenerations of IEPs over evolution, thus they lost the ability to mobile and promote intron splicing [10]. So far, only one chloroplast group II intron within the *trnK* gene encodes a maturase, denoted MatK [12,13]. Certain group I introns are mobile genetic elements since they encode homing endonucleases (HEs). These HEs are site-specific DNA endonucleases that function in intron mobility and in some cases function in RNA splicing [14]. Only one group I intron in *trnL* gene is present in land plant chloroplasts; however, it fails to self-splice [15]. Therefore, the splicing of group I and group II introns in land plant chloroplasts relies on nuclear-encoded splicing factors. This review will discuss recent advances on the splicing of chloroplast introns in land plants, with emphasis on the splicing factors and their possible roles in RNA splicing.

## Chloroplast introns

The presence of introns in chloroplast genes is a feature of the chloroplast genome. [6,8,12,16]. There are 17 to 20 group II introns and only one group I intron in *trnL* (*UAA*) in land plant chloroplast genomes. The basic set of these 20 introns (*clpP-2* excluded) is also shared among bryophytes, indicating that these chloroplast introns were acquired before the emergence of land plants [17–20].

### Group I introns

The structure of group I introns is relatively small and uniform. Each group I intron is folded into a secondary structure with 10 paired domains, P1 to P10, which then fold to form a tertiary structure with three domains [21]. Each domain functions specifically in RNA folding. Two helical domains constitute the central catalytic core of group I introns, which are stabilized via peripheral domains [8,22]. The splicing of group I introns is accomplished by a two-step *trans*-esterification reaction, first at 5’ and then at the 3’ splice sites. Firstly, exogenous guanosine attacks the 5’ splice site and attaches to the 5’ end of the intron, releasing the 5’ exon. Secondly, 3’-OH of the released exon attacks the 3’ splice site ligating the exons and releasing the intron. The prerequisite of successful splicing is the correct folding of the introns [1,8,16].

Group I introns are mobile genetic elements. This means that they can insert into other intronless genes, thus resulting in their spread [8]. The mobility of group I introns is accomplished by intron homing and reverse splicing [1]. Reverse splicing is the process by which an intron is integrated into other RNA molecules. Till now, no direct evidence is available on the spread of group I introns via reverse splicing. However, certain experimental and comparative data support this theory [8].

Intron homing refers to a process through which an intron spreads into the homologous position in an intronless allele. This process is catalysed by homing endonucleases (HEs), which are encoded by homing endonuclease genes (HEGs) within introns [8,23]. The HEG will lose its function of promoting intron mobility when the intron is immobilized in a population, and the HEG will be lost over time. Thus, HEG is absent in most of the group I introns [8,23]. Based on conserved amino acid motifs, HEs can be categorized into four families: LAGLIDADG, HNH, GIY-YIG, and His-Cys box [21,23,24]. Numerous HEs also possess maturase activity, which is postulated to be acquired to compensate for the reduced splicing efficiency caused by the presence of HEG [25]. As autocatalytic ribozymes, most group I introns have retained self-splicing ability. For instance, several group I introns in *C. reinhardtii* chloroplasts and other algae have been detected to have the ability of self-splicing *in vitro* [15,26]. Nevertheless, the only group I intron within the *trnL* gene in land plant chloroplasts has lost this ability, thus its splicing requires the assistance of additional proteins.

### Group II introns

Group II introns are primarily present in eukaryotic organellar and bacterial genomes and rarely in eukaryotic nuclear genomes [9]. Group II introns in organelles may have degenerations in IEPs and RNA structures over time, such as mispairs in domains V and VI, or large insertions or deletions in regions that are vital for catalysis of splicing, which result in the introns’ inability to self-splice and loss of mobility [10,12,27–29]. Although group II introns do not contain a conserved primary nucleotide sequence, their secondary structure is conserved.

Each group II intron consists of 6 paired domains, domains I to VI, radiating from a central core [10,11,30,31]. Domain I is the largest domain of group II intron, it plays a role in recognizing and positioning the exon for catalysis due to the presence of two exon binding sites (EBS1 and EBS2). These EBS can form 5 ~ 6 base pairs with corresponding intron binding sites 1 and 2 (IBS1 and IBS2) located at the 3’ end of the upstream exon [9]. Domain II can recruit domain III into the core of intron RNA and contribute to RNA folding and enhance catalytic activity of intron RNA. The domain IV loop encodes IEP, which functions as a maturase that can bind to unspliced introns and change its conformation to facilitate splicing of the intron. Domain V is small and highly conserved in structure, and it contains a 34 nt long sequence that is part of the catalytic core, and thus the domain V also participates in ribozyme activity. Domain VI contains the branch point adenosine required for the first *trans*-esterification reaction [9–11,32]. Although group II introns possess conserved secondary structures, based on their structural features and mechanisms of exon recognition, they are subdivided into three subclasses: IIA, IIB, and IIC. IIC introns are found only in bacteria, and IIA and IIB only in plant organelles [11,31].

The splicing of group II introns is also accomplished by a two-step *trans*-esterification reaction, which is different from group I introns. Firstly, instead of an exogenous guanosine, the 2’-OH of a bulged adenosine within domain VI attacks the 5’ splice site and attaches to the 5’ end of the intron. This results in the release of the upstream exon and the formation of lariat intermediate. Secondly, the 3’-OH of the released upstream exon attacks the 3’ splice junction, yielding ligated exons and an excised intron in a lariat form [9,11,12,21].

Previous studies on the structure and splicing mechanism of each intron type have revealed that group II introns are closely related to spliceosomal introns [11,33–35]. The major similarities between the two intron types are their similar splicing mechanisms, both of which undergo a two-step transesterification reaction and form a lariat intermediate, and similar structures and functions of their active sites in the catalysis of splicing [31]. Besides, the similarities between group II intron reverse transcriptases (RTs) and the spliceosomal protein pre-mRNA processing protein 8 (Prp8) also support the notion that the spliceosome was derived from group II introns [36–38]. Prp8, located at the centre of the spliceosome, is an integral part of the U5 snRNP [38]. Due to the sequence and structure similarities between one of the Prp8 domains and a bacterial group II intron RT, Prp8 was considered to have evolved from the reverse transcriptase [37,38]. The crystal structure of Prp8 revealed that several domains, including the RT domain of Prp8, form a large cavity, which can accommodate the catalytic centre of group II intron RNA [37,38]. Like many group II introns, which do not have the endonuclease domain, Prp8 also lacks an active endonuclease domain [38]. The telomerase and group II introns perform telomere addition and intron mobility, respectively, via a target-primed reverse transcription (TPRT) mechanism. Thus, telomerase reverse transcriptase (TERT) also favours spliceosome origin from group II introns [39]. Thus, it is also believed that eukaryotic spliceosomes could have evolved from group II introns [11,31].

## Splicing factors in land plant chloroplasts

As described above, the efficient splicing of degenerate group I and group II introns in land plants requires additional proteins. About twenty group II introns and one group I intron present in land plant chloroplasts have lost self-splicing ability and thus require additional splicing factors. These splicing factors can be categorized into two classes, maturase, and nuclear-encoded proteins. Their roles are summarized in Table S1. Maturases are encoded within the introns, and they have dual functions both in intron mobility and intron splicing. Nuclear-encoded splicing factors are recruited more recently from the nuclear genome.

### Maturases

Maturases are intron-encoded proteins that are implicated in the splicing of their host intron [9,40,41]. Maturases aid intron splicing by helping folding the RNA into a catalytically active conformation or stabilizing the active structure [9,41]. Intron-encoded proteins (IEPs) are characterized into four domains: a) RT domain involved in intron mobility, b) maturase (also called X) domain involved in RNA binding and intron splicing, c) DNA binding domain (DBD), and d) endonuclease domain involved in retrotransposition [28,38]. IEPs are dual-function proteins that are involved in intron mobility and RNA splicing, and their role in splicing is known as maturase activity [11]. As per the previous hypothesis, IEPs were involved in intron mobility, and during evolution, they acquired maturase activity to reduce the adverse consequences of endonuclease gene invasion on intron self-splicing [25,42]. Based on previous studies on bifunctional proteins that act on both DNA and RNA, for instance, the study on *E. coli* maturase I-AniI, it seems likely that the maturase function was derived from its novel binding potential to RNA template [10,25].

Most group I intron-encoded maturases belong to the LAGLIDADG class of homing endonucleases, and some of these proteins lack DNA endonuclease activity and retain only the maturase activity [43]. *trnL*, the only group I intron in land plant chloroplasts, does not encode a maturase. There is only one group II intron-encoded maturase in land plant chloroplasts, denoted MatK, which is encoded by group II intron within *trnK* [12]. MatK contains a degenerate RT domain and lacks the endonuclease domain, but have a X domain, which shows that it has retained an essential splicing role [9]. An RNA immunoprecipitation assay in tobacco showed that MatK could capture seven chloroplast group II introns including the *trnK* intron [13]. In addition, the absence of MatK in barley mutant deficient in chloroplast ribosomes is related to the failure in splicing of *trnK* and other six introns [44]. These findings indicate that MatK is required for the splicing of the *trnK* intron, and the other six introns [13,44].

Four nuclear-encoded maturases (nMATs) have been found in *Arabidopsis* [45], three (nMAT1-3) of which are located in mitochondria and one (nMAT4) is dual localized in chloroplasts and mitochondria [46]. These nuclear-encoded mMATs probably derived from group II introns [9]. Similar to MatK, these nMATs all contain a conserved X domain. nMAT1 and nMAT2 have degenerate RT domains and lack the DBD and endonuclease domains. Interestingly, nMAT3 and nMAT4 contain all domains but have lost the endonuclease activity due to mutations [28]. In *Arabidopsis*, previous studies have shown that nMAT1, 2, 4 all participate in the splicing of several mitochondrion group II introns [46–49]. In maize, nMAT3 is required for the splicing of several mitochondrion group II introns [50]. Therefore, no nuclear-encoded maturases have been found to participate in intron splicing in land plant chloroplasts.

### Nuclear-encoded splicing factors

**Figure 2.**
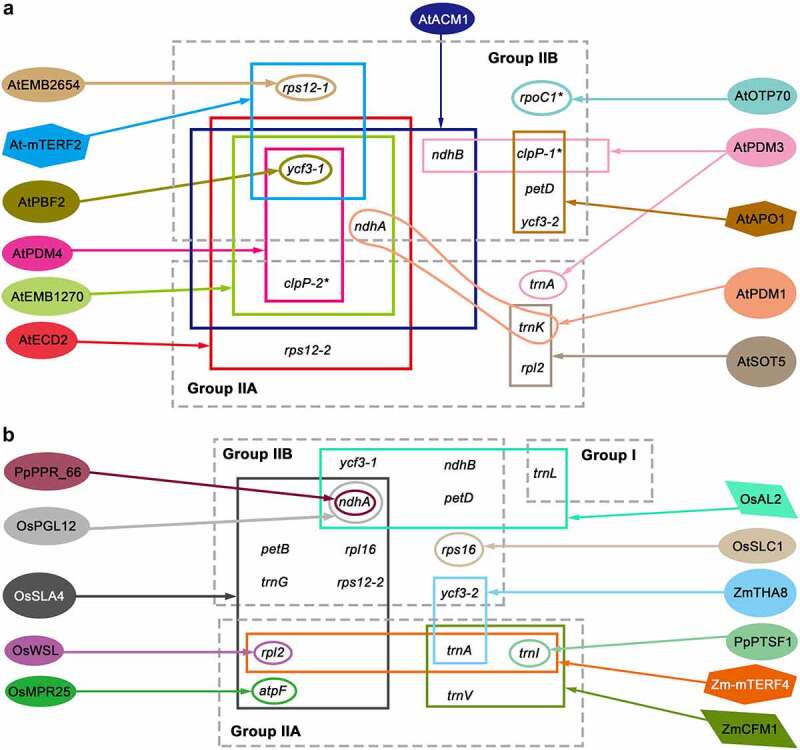
Nuclear-encoded proteins that promote chloroplast intron splicing in *Arabidopsis* (a) and other land plants (b). Most of the splicing factors that have been diagrammed in previous reviews (see de Longevialle et al., 2010; Stern et al., 2010; Khrouchtchova et al., 2012; Germain et al., 2013) are not shown in this figure. The chloroplast introns are classified into group I, IIA, and IIB. The oval represents PPR proteins, the quadrilateral represents CRM domain proteins, and the hexagon represents other domain proteins. The asterisk refers to introns present in *Arabidopsis* but absent in rice and maize.

Dozens of nuclear-encoded proteins from different protein families have been identified to participate in the splicing of chloroplast introns (see Fig. 2). Most of these splicing factors are RNA-binding proteins, some of which have long been reported to have RNA-binding domains, such as pentatricopeptide repeat (PPR) proteins, while others were first identified as RNA-binding proteins only by discovering their involvement in RNA splicing processes, such as proteins harbouring CRM, RNase III, PORR, Whirly, or DUF794 domains [51–55].

### PPR domain family

PPR protein family is a major group of proteins involved in organellar RNA splicing. PPR proteins are typically defined as tandem arrays of the approximately 35-amino-acid long repeat (PPR motif), and each member of the PPR protein family consists of 2 to 30 PPR repeats [56]. The PPR protein specifically binds to its RNA target based on the one-repeat:one-nucleotide recognition mechanism [57–60]. PPR proteins are widely distributed in chloroplasts, mitochondria or both of eukaryotes, especially in land plants and moss, but less in other eukaryotes, such as algae and animals [61].

PPR proteins are divided into P and PLS subfamilies based on the characteristics of their PPR repeats. The P-class PPR proteins consist of tandem arrays of only P repeats, while PLS-class proteins contain triplets of P, L (Long, generally 36 amino acids), and S (Short, generally 31 amino acids) repeats [56,62,63]. Some P-class proteins also have extra domains other than P repeats at their C-terminal, such as the small MutS-related (SMR) domain [64]. PLS-class PPR proteins are further divided into four subgroups (denoted PLS, E, E+, and DYW) based on their C-terminal domains [62,65]. These domains are closely related to RNA editing in organelles [66]. On the other hand, P-class proteins are required for diverse RNA maturation processes, including RNA stability, intergenic RNA cleavage, and RNA splicing [62].

Till now, around twenty PPR proteins have been reported to participate in RNA splicing in land plant chloroplasts, and most of these PPR proteins belong to the P subfamily. Moreover, most of these PPR proteins splice one or two specific intron targets. For instance, *Arabidopsis* Organelle Transcript Processing 51 (OTP51), containing seven PPR motifs and two LAGLIDADG motifs, is primarily required for the splicing of *ycf3* intron 2 [67]. Zm-OTP51 was shown to bind the first 197 nt of this intron with high affinity [68]. Maize THYLAKOID ASSEMBLY 8 (THA8) is a PPR protein with only four PPR motifs and no other domains [68]. RIP-chip assay and slot-blot hybridizations demonstrated that THA8 is responsible for the splicing of maize *ycf3* intron 2 and *trnA* intron, a conserved function in *Arabidopsis*. Weak binding of recombinant THA8 to the first 197 nt of *ycf3* intron 2 was detected via electrophoretic mobility shift assays (EMSAs). The results of this assay were in line with the short PPR repeats of THA8 [68]. Bioinformatics and structural analyses identified that *Brachypodium distachyon* THA8 dimer binds to two RNA fragments in *ycf3* intron 2, moreover, it is the binding of RNA that induces the dimerization of THA8 [69]. PpPPR_66, a *Physcomitrella patens* P-class PPR protein, is implicated in the *ndhA* intron splicing, it has been demonstrated to bind to the 115-nt region extending from part of exon 1 to the *ndhA* intron. In addition, the splicing of *ndhA* pre-mRNA was also defected in *Arabidopsis* [70]. Notably, the recognition sequence of PpPPR_66 predicted by the PPR code is not within *ndhA* intron, suggesting that PpPPR_66 might recognize the RNA structure [70]. In the *in vitro* analysis, OTP51, THA8, and PpPPR_66 were found to bind to RNA; however, their precise binding sites should be determined via further analyses, such as RNA footprint analysis and EMSAs. Fortunately, four P-class PPR proteins from different species have defined their precise binding site in introns. For instance, in *Arabidopsis*, the qRT-PCR analysis revealed that EMB2654 is involved in *rps12* intron splicing. Furthermore, RNA footprint analysis and EMSAs revealed that EMB2654 binds directly to the 3’ end of *rps12* intron 1a [71,72]. Maize PPR4, which contains 16 PPR motifs and an RNA recognition motif (RRM), has been demonstrated to be involved in the *trans*-splicing of *rps12* intron 1 via a series of biochemical assays [73]. Further studies have shown that PPR4 plays the same role in *Arabidopsis* and rice [72]. *ppr4* mutants in *Arabidopsis* and rice are embryo-lethal and seedling-lethal, respectively, suggesting that PPR4 is indispensable for the development and growth of both dicot and monocot plants. RNA coimmunoprecipitation (RIP) assay combined with RNA footprint analysis and EMSAs revealed that PPR4 could bind directly to the 5’ end of *rps12* intron 1b [72]. The binding of PPR4 and EMB2654 to *rps12* introns 1b and 1a, respectively, may have altered the structure of domain III within the intron, causing it to fold into a structure that facilitates effective *trans*-splicing of *rps12* intron 1 [72]. In moss *Physcomitrella patens*, a P-class PPR protein PpPPR_4, also named plastid tRNA splicing factor 1 (PpPTSF1), was shown to be responsible for the splicing of pre-tRNA^Ile^. EMSA revealed that recombinant PpPPR_4 binds to a 25-nt region in domain III of the tRNA^Ile^ intron [74]. qRT-PCR and RT-PCR analyses revealed that *Arabidopsis* PPR protein PHOTOSYSTEM I BIOGENESIS FACTOR2 (PBF2) is responsible for the splicing of *ycf3* intron 1. Prediction of recognition sequence, bioinformatics analysis as well as EMSA showed that PBF2 bound to a specific sequence within intron 1 of *ycf3* [75]. Unlike those PPR proteins characterized above, which have been shown to bind intron RNA *in vitro*, some P-class proteins reported to participate in RNA splicing have not. For example, *Arabidopsis* SUPPRESSOR OF THF1 5 (SOT5) is a PPR protein with 11 PPR repeats, prediction of binding target combined with qRT-PCR and RT-PCR assays have identified that SOT5 is implicated in the splicing of chloroplast *rpl2* and *trnK* introns [76]. SOT5 is predicted to bind to a specific sequence in *rpl2* and *trnK* introns, but it has not been demonstrated to bind intron RNA *in vitro* as the expression of recombinant SOT5 is not available. In rice, *OsPPR6* is involved in splicing *ycf3* introns, but which one is not determined. Besides, it also participates in editing of the *ndhB* transcript [77]. The *Oryza sativa* SEEDLING-LETHAL CHLOROSIS 1 (OsSLC1) contains 12 PPR motifs, RT-PCR and qRT-PCR data demonstrated that it mainly participate in the intron splicing of *rps16* in rice [78]. However, more biochemical evidence is required to support this hypothesis; however, it is challenging to isolate functional PPR protein *in vitro*.

Certain P-class PPR proteins affect multiple intron targets, unlike the above-mentioned P-class PPR proteins that affect one or two introns. Among them, a recently reported *Arabidopsis* P-class PPR protein, EMB1270, cooperate with CFM2 to promote the splicing of *ycf3* intron 1, *clpP* intron 2, and *ndhA* intron. Moreover, EMSA analysis demonstrated the direct binding of truncated EMB1270 with these three introns containing predicted consensus sequences [79]. Pigment-Defective Mutant3 (PDM3) is a P-class PPR protein harbouring 12 PPR motifs, it might be involved in the intron splicing of *ndhB, trnA*, and *clpP* intron 1 in *Arabidopsis* [80]. *Arabidopsis* PDM4 is implicated in the splicing of *ycf3* intron 1 and *clpP* intron 2 [81,82], Albino Cotyledon Mutant1 (ACM1) in *ndhA* intron, *ndhB* intron, *clpP* intron 2, and *ycf3* intron 1 [83], Early Chloroplast Development 2 (ECD2) in *ndhA* intron, *rps12* intron 2, *ycf3* intron 1, and *clpP* intron 2 [84], rice WHITE STRIPE LEAF 4 (WSL4) in *rps12* intron 2, *ndhA, atpF*, and *rpl2* introns [85]. Another rice P-class PPR protein WHITE STRIPE LEAF 5 (WSL5) harbours 15 PPR motifs and an RRM motif at its N-terminal. According to a previous report, it affects the splicing of *rpl2* and *rps12* intron 2, and also the editing of two editing sites [86]. In maize, EMB-7 L can splice *rps12* intron 1, *rpl2, atpF*, and *ndhA* [87]. EMB-7 L is predicted to target *rps12* intron 1 based on PPR code, that is, the 5th and 35th amino acids of each PPR motif determine its recognition specificity of nucleotides [57–60]. But biochemical data *in vivo* and *in vitro* are needed to identify the target intron of EMB-7 L. The splicing function of most of these proteins was validated via RT-PCR and qRT-PCR evidence, more biochemical data are required to identify their specific intron targets. These PPR proteins affecting multiple introns might recognize the RNA structure specific to these introns, or bind to the common sequence of their target introns, or alternatively, one PPR protein just directly targets one specific intron, and other introns it affects are direct targets of other proteins associated with it. It remains to be tested which hypothesis is reliable.

The above-mentioned PPR proteins involved in RNA splicing belong to the P subfamily. In addition, certain PLS-class PPR proteins are also reported to participate in RNA splicing, and most of them target a single intron. Among them, some have lost the ability of RNA editing but gained the ability to splice. For example, the *Arabidopsis* ORGANELLE TRANSCRIPT PROCESSING 70 (OTP70) with an E domain at its C-terminal is a member of the E-subgroup PPR proteins. E domain has been proved to be indispensable for RNA editing by deletion assays [88,89]. However, the E domain of OTP70 is truncated and has been validated to lack the ability of RNA editing. Nonetheless, OTP70 is identified to be responsible for the splicing of *rpoC1* intron, and this function is independent of the E domain [90]. WHITE STRIPE LEAF (WSL) is a rice PPR protein belonging to the PLS subfamily, and notably, it consists of 14 PPR motifs and no other functional domains, such as E, E+ or DYW at its C-terminal. Studies showed that WSL is responsible for the splicing of chloroplast *rpl2*, and has no effect on RNA editing [91]. Rice PLS-class PPR protein Seedling-Lethal Albino 4 (SLA4) has 15 PPR motifs and an atypical DYW-like motif, it was reported to affect the intron splicing of *petB, ndhA, atpF, rpl2, rpl16, trnG*, and *rps12* intron 2, but have no effect on RNA editing [92]. PALE-GREEN LEAF12 (PGL12) is also a PLS-class PPR protein with no functional domain at its C-terminal, findings showed that it was not implicated in RNA editing, but in the splicing of *ndhA* transcript in rice [93]. There are also PLS-class PPR proteins play roles in both RNA editing and intron splicing. *Arabidopsis* Pigment-Deficient Mutant 1/ Seedling Lethal1 (PDM1/SEL1), as a PLS-class PPR protein, lacks the E, E+ or DYW domain at its C-terminus that is essential for RNA editing, but it is still demonstrated to affect RNA editing of *accD* [94]. In addition, PDM1 was reported to participate in splicing *ndhA* and *trnK* introns [95]. *Arabidopsis* ATPF EDITING FACTOR 1 (AEF1) and its rice orthologue MITOCHONDRIAL PPR25 (MPR25) are PLS-class PPR proteins with an E domain at their C-terminal. They are reported to be implicated in RNA editing, as well as *atpF* splicing in *Arabidopsis* and rice [96].

Two other PPR proteins in chloroplasts, HCF152 and PPR5, might be responsible for RNA splicing. *Arabidopsis* HCF152 is a PPR protein composed of 12 PPR repeats. It participates in the processing of chloroplast *psbB-psbT-psbH-petB-petD* polycistronic transcripts [97]. Previous studies have shown reduced accumulation of spliced *petB* intron in *hcf152* mutant. However, HCF152 is identified to bind specifically to the untranslated region between *psbH* and *petB*. Therefore, HCF152 may participate directly in splicing *petB* intron, or alternatively, it may participate in stabilizing the splicing products [98]. In maize, PPR5 is responsible for stabilizing the unspliced precursor of *trnG*-UCC, as it binds to this region *in vivo* [99]. PPR5 may also participate in splicing *trnG*-UCC directly *in vitro*, as its binding site in *trnG*-UCC intron contains two crucial group II functional elements [100].

### CRM domain family

Another protein family involved in intron splicing is the protein family containing the chloroplast RNA splicing and ribosome maturation (CRM) domains. The CRM domain originated from an ancient ribosome-associated protein, it exists as a stand-alone protein in Archaea and Bacteria, and the CRM proteins have developed multiple CRM domains in plants [51]. The previous study in maize has shown that the CRM domain can bind RNA *in vitro*, and a conserved ‘GxxG’ motif contributes to its RNA binding activity [51]. So far, all the characterized CRM proteins are required for RNA splicing in land plant chloroplasts or mitochondria.

There have been 10 CRM domain proteins identified to participate in RNA splicing in maize, *Arabidopsis*, and rice: Chloroplast RNA Splicing 1 (CRS1), CRS2-associated factors 1 (CAF1), CAF2, CRM Family Member 2 (CFM2), CFM3, CFM1, and their orthologs. Most CRM proteins are initially studied in maize and *Arabidopsis*, and then characterized in rice. Their functions in maize and *Arabidopsis* are almost conserved, but distinct in rice. Maize CRS1 was the first characterized CRM protein, it contains three CRM domains [101,102]. CRS1 is involved in the splicing of *atpF* intron (group IIA) [102]. Futher studies revealed that CRS1 binds to two non-conserved intron segments in domains I and IV of *atpF* intron *in vitro*, this further demonstrated that CRS1 is involved solely in the splicing of *atpF* intron [103]. Unlike the splicing function of orthologous CRS1 in maize, rice protein OsAL2 is not only involved in the splicing of four group II introns, including *ndhA, petD, ndhB*, and *ycf3* intron 1, but also the group I intron, *trnL* [104]. CRS2 is closely related to peptidyl-tRNA hydrolases (PTHs), but has lost the PTH activity due to the substitution of several amino acids [105]. CRS2 is responsible for the splicing of several group II introns, and generally, introns requiring CRS2 to assist splicing also require the involvement of CAF1 and CAF2 [106]. CAF1 and CAF2 are identified through yeast two-hybrid screen using CRS2 as bait. The two proteins can interact with the CRS2 protein respectively, forming two complexes CRS2-CAF1 and CRS2-CAF2. In maize, the CRS2-CAF1 complex is involved in the intron splicing of *petD, trnG, rps16, rpl16, ndhA*, and *ycf3* intron 1. Besides these introns, *Arabidopsis* CAF1 is also involved in the intron splicing of *rpoC1* and *clpP* intron 1, two introns absent in maize [106,107]. The CRS2-CAF2 complex is involved in the splicing of a subset of group IIB introns, including *petB, ndhB, ndhA, rps12* intron 1 and *ycf3* intron 1 [106]. CRM Family Member 2 (CFM2) is another CRM protein with four CRM domains. RIP-chip assay combined with coimmunoprecipitation and cosedimentation assays in maize together showed that CFM2 and CAF1 and/or CAF2 are present in large ribonucleoprotein particle (RNP) complexes that contain *trnL* intron, *ndhA* intron and *ycf3* intron 1. In addition, the *Arabidopsis* AtCFM2 also influences the splicing of *clpP* intron 2, which is absent in maize [108]. Unlike its orthologs in *Arabidopsis* and maize, rice proteins OsCAF1 and OsCAF2 are implicated in the splicing of group IIA as well as IIB introns. Similarly, both OsCAF1 and OsCAF2 interact with OsCRS2 to form a complex, respectively [109,110]. OsCFM2 in rice participates in the splicing of one group I intron, *trnL*, and five group II introns, including *rps12, rpl2, ndhA, atpF*, and *ycf3* intron 1 [111]. CFM3, harbouring three CRM domains, is implicated in splicing introns of *rps16, rpl16, ndhB, petB, petD*, and *trnG* in *Arabidopsis*, and *rps16, rpl16, petD*, and *ndhB* in rice [112]. Thus, the introns that require CRS2/CAF complexes for their splicing also require CFM2 or CFM3, but not both. CFM1 is a recently characterized CRM domain protein in *Setaria viridis* and maize [113]. RIP-chip data demonstrated that Zm-CFM1 promotes the intron splicing of *trnA, trnI* and *trnV*, and these introns have not been shown to require any previous reported CRM domain proteins. The function of CFM1 is conserved in *Setaria viridis* and maize, as the splicing defects of these introns were also identified in *Sv-cfm1* [113]. In conclusion, the CRM proteins function in splicing of almost all the chloroplast introns, and each subset of introns spliced by each CRM protein is overlapping but distinct.

### Other domain proteins

In addition to the PPR and CRM protein families, some proteins containing other domains also are reported to participate in chloroplast intron splicing. RNC1 was identified in CAF1 and CAF2 coimmunoprecipitates through mass spectrometry analysis [54]. RNC1 has two ribonuclease III (RNase III) domains, but lacks endonuclease activity due to the loss of several essential amino acids. It has been identified that RNC1 promotes the splicing of a subset of group IIA introns, including *rps12* intron 2, *atpF, trnK, trnV, trnI, trnA* introns, and group IIB introns, including *petB, petD, ndhB*, and *trnG* introns [54]. Moreover, recombinant RNC1 can bind RNA with high affinity *in vitro*, indicating that RNC1 is an RNA binding protein, and it may promote intron splicing via binding directly to RNA [54]. What’s This Factor? (WTF1) is another splicing factor identified from CAF1 and CAF2 coimmunoprecipitates through mass spectrometry analysis [52]. It has been shown that WTF1 and RNC1 form a heterodimer to facilitate splicing most of chloroplast group II introns. WTF1 has a plant-specific domain of unknown function 860 (DUF860), also known as Plant Organelle RNA Recognition (PORR). Both WTF1 and DUF860 can bind RNA *in vitro*, suggesting that DUF860 is a newly identified RNA-binding domain [52]. Thus, DUF860 expands the repertoire of RNA-binding domains specific to plants. Maize ZmWHY1 is a member of the Whirly protein family, which is plant-specific. ZmWHY1 associates with CRS1 to facilitate the splicing of *atpF* intron. EMSAs demonstrated that ZmWHY1 can bind single-stranded DNA and RNA [53]. ACCUMULATION OF PHOTOSYSTEM ONE1 (APO1) was initially thought to promote the assembly of [4Fe-4S] cluster-containing chloroplast complexes, such as the photosystem I (PSI) and NADH dehydrogenase complexes [114]. In maize, APO1 coimmunoprecipitated with CAF1, which led to the speculation of the involvement of APO1 in intron splicing. Subsequent studies have shown that APO1 promotes the splicing of *ycf3* intron 2, *petD* intron, and *clpP* intron 1 [55]. APO1 contains an unknown functional domain DUF794, which is a plant-specific domain containing two motifs that resemble zinc fingers. EMSAs showed that maize and *Arabidopsis* recombinant APO1 binds RNA in *ycf3* intron 2 with high affinity [55]. Thus, DUF794 was termed as an RNA-binding domain. RH3 in maize and *Arabidopsis* is a member of the DEAD box RNA helicase family. It is responsible for the intron splicing of *trnA, trnI, rpl2*, and *rps12*, and may also contribute to 50S ribosome biogenesis [115]. In addition, one later study demonstrated that AtRH3 also participates in splicing *trnL, trn*K, and *atpF* introns [116]. The mitochondrial transcription termination factor (mTERF) protein family in metazoan mainly affects mitochondrial transcription, ribosome biogenesis and DNA replication [117]. Maize Zm-mTERF4 is a member of this family, and it was identified to participate in the intron splicing of *trnI*-GAU, *trnA*-UGC and *rpl2* [118]. Another mTERF protein in *Arabidopsis*, mTERF2, was shown to participate in the splicing of *ycf3* intron 1 and *rps12* intron 1 through RNA-seq and RNA gel-blot analyses [119]. These findings extend the functional repertoire of mTERF family in plants. Taken together, characterizations of these domains derived from diverse protein families extend the repertoire of RNA-binding domains. Some of these protein factors have lost their ancestral activities, and they are recruited to participate in RNA splicing, such as RNC1. While some splicing factors might also have DNA binding activity, such as WHY1. These proteins were demonstrated to be associated with RNAs, but not specifically with a certain intron. Thus, maybe these proteins are recruited to participate in splicing via protein–protein interactions and they function in intron splicing together with other splicing factors.

## The roles of splicing factors in RNA splicing

Although lots of RNA-binding proteins have been identified to participate in chloroplast intron splicing in land plants through reverse genetic screen or coimmunoprecipitation assay combined with mass spectrometry analysis, the specific roles of these splicing factors during RNA splicing remain ambiguous. The possible roles of splicing factors are speculated based on existing evidence. The common feature of these splicing factors including matuarases is that most of them are bifunctional moonlighting proteins, since they participate in other cellular functions besides serving as splicing factors. For instance, bacterial peptidyl-tRNA hydrolase-related protein CRS2 moonlights as a splicing factor. WHY1, which belongs to a family that has been described as DNA-binding proteins also moonlights as a splicing factor. Similarly, RNC1 with two RNase III domains also was recruited to function in splicing. Maturases have dual functions both on DNA and RNA. In addition, many factors have been identified based on genetic screens or limited biochemical assays, and they affect multiple introns, but their exact role is not clear, these proteins may have other important functions, and maybe they just moonlight as a splicing factor, or maybe they are recruited via protein–protein interactions and function in splicing together with other splicing factors [9].

Given previous findings, each organellar intron requires more than one splicing factor, and these splicing factors are found in one or more intron-containing RNP complexes. Cosedimentation via a sucrose gradient assay showed that THA8 is present in large RNP complexes containing *trnA* intron and *ycf3* intron 2. In addition, the communioprecipitation assay showed that THA8 associates with WTF1 and RNC1 [68]. Therefore, it is likely that THA8 associates with WTF1/RNC1 heterodimer to advance the splicing of their shared intron target *trnA*. Meanwhile, THA8 may also cooperate with OTP51 and APO1 to facilitate the splicing of their shared intron target, *ycf3* intron 2. Sucrose gradients and coimmunoprecipitation assays showed that CFM2 is associated with CAF1, CAF2, *ndhA* intron, *ycf3* intron 1, and *trnL* intron in large chloroplast stromal RNP complexes. The intron splicing of *ndhA* requires CFM2 and CRS2/CAF2 complex, and the splicing of *ycf3* intron 1 requires CFM2, CRS2/CAF1, and CRS2/CAF2 complexes [108]. Cosedimentation and coimmunoprecipitation data validated that CFM3 coimmunoprecipitated with CAF1, CAF2, and RNC1, and these splicing factors associated simultaneously with their shared intron targets in large RNPs [112]. The splicing of *atpF* intron requires CRS1, ZmWHY1, WTF1 and RNC1, and CRS1 interacts with ZmWHY1 in an RNA-dependent manner [52–54,102]. WTF1 coimmunoprecipitated with CRS1, CAF1, CAF2 and RNC1, and they are found in stromal RNP complexes of ~ 600 to 700 kD including a set of group II introns [52]. The *trans*-splicing of *rps12* intron 1 requires PPR4, EMB2654, and CRS2/CAF2 complex [71,72]. Thus, there are diverse chloroplast group II intron RNP complexes containing several splicing factors and a set of introns to advance the splicing of distinct group II introns, and the roles of these splicing factors are considered to assist the intron folding into its catalytically active structure favourable for splicing by stabilizing its inherently native structure, preventing the formation of nonnative structures, or destabilizing nonnative structures [54].

Nonetheless, the precise function of these splicing factors in intron folding or sequence recognition in the process of splicing remains unclear. This is due to the lack of identification of precise and direct binding sites of most splicing factors. Identifying specific binding sites of some splicing factors could improve our current understanding of their roles in RNA splicing. CRS1 was the first protein explored to reveal its direct binding site. *In vitro* biochemical analyses have shown that CRS1 facilitates the correct folding of *atpF* intron through binding with its two regions in domains I and IV [103]. Later, PPR proteins OTP51, THA8, PTSF1, PpPPR_66, EMB2654, PPR4, and PBF2 were also shown to bind RNA directly *in vitro*. For instance, OTP51 and THA8 were shown to bind the first 197 nt of *ycf3* intron 2 with high and weak affinity, respectively [68]. PTSF could bind to a 25-nt region within the domain III of tRNA^Ile^ intron, forming a nanoloop [74]. Domain III functions as a catalytic effector, thus, the function of PTSF might promote the interaction of domain III with other domains PpPPR_66 binds preferentially to a 115-nt region at the 5’ half of the domain I of the *ndhA* intron, and this region encompasses from part of exon 1 to the intron, which can form a long stem-loop structure [70]. EMB2654 and PPR4 were demonstrated to bind to *rps12* intron 1a and 1b, respectively, which is speculated to help the folding of domain III of *rps12* intron 1 [72]. PBF2 was shown to bind a specific sequence within domain II of *ycf3* intron 1 [75]. Although almost all the splicing factors are considered RNA-binding proteins, only PPR proteins were validated to bind RNA directly *in vitro* using RNA footprinting analysis and EMSAs. Moreover, most PPR proteins are specific to only one target intron, which was determined using the structure and PPR codes of PPR motifs, and the CRM domain does not possess this specificity for RNA. Since each intron generally requires several splicing factors, we speculate that splicing factors in intron splicing are involved in the binding of PPR protein to its target intron with sequence specificity. Thus, they assist the intron in folding into a structure necessary for recruiting relevant general splicing factors, such as CAF1 and CAF2. Also, some general splicing factors are recruited by the PPR protein via direct protein–protein interactions. These splicing factors as well as their shared introns form RNP complexes to promote the intron splicing. Nevertheless, further studies are needed to test this hypothesis in the future.

## Conclusions and prospects

RNA splicing is a complex process involving multiple splicing factors from diverse protein families. Some of these splicing factors are inherently considered to be RNA-binding proteins, such as PPR proteins, and others (CRM, PORR, RNase III, APO protein families, and so on) are first identified as RNA-binding proteins by identifying their functions in chloroplast RNA splicing. Diverse splicing factors are involved in the splicing of distinct but overlapping intron subsets. However, most PPR proteins are implicated in splicing single introns as they bind RNA in a sequence-specific manner. Several splicing factors, which share at least one intron target, cooperate to form splicing complexes, the RNP complexes, which assist intron folding and promote intron splicing. However, only a few PPR proteins have identified the exact binding sequence within intron domains. Thus, the precise role of each splicing factor within RNP complexes in facilitating splicing is still unclear. Crystal structure analysis and prediction of recognition sequences by PPR code and bioinformatics analysis will be needed to obtain precise binding sequences of more splicing factors. Furthermore, *in vivo* and *in vitro* biochemical data are also required to determine whether splicing factors with only RT-PCR and qRT-PCR evidence are directly involved in intron splicing. Thus, it seems likely that PPR proteins specifically binding to intron target are more likely involved in splicing directly, and proteins affecting multiple introns are more likely working with other proteins and having an indirect involvement in splicing. In the future, more potential splicing factors can be screened by reverse genetic screening of mutants or mass spectrometry analysis after coimmunoprecipitation with known splicing factors. All of these investigations can contribute to the clarification of the splicing mechanism of splicing factors.

## Supplementary Material

Supplemental Material
