# Supplementary material for: An overview of RNA splicing and functioning of splicing factors in land plant chloroplasts
Source: RNA Biol. 2022 Jul 11;19(1):897–907. doi: 10.1080/15476286.2022.2096801 (PMC9275481; doi:10.1080/15476286.2022.2096801)
Supplement: Supplemental Material [file KRNB_A_2096801_SM4683.pdf]

Table S1. Plastid and nuclear-encoded factors involved in the splicing of chloroplast introns.

Type	Name	A.thaliana	O.Sativa	Affected introns	References
Maturases	matK			*trnK* intron, *atpF* intron, *trnA* intron, *trnI* intron, *trnV* intron, *rpl2* intron, *rps12* intron 2	Zoschke et al., 2010
PPR proteins	AtOTP51	At2g15820	Os02g47360	*ycf3* intron 2	de Longevialle et al., 2008; Khrouchtchova et al., 2012
	ZmTHA8	AT3G27750	Os04g39970	*ycf3* intron 2; *trnA* intron	Khrouchtchova et al., 2012; Ke et al., 2013
	PpPPR_66	At2g35130	Os02g07360	*ndhA* intron	Ito et al., 2018
	AtEMB2654	At2g41720	Os07g11280	*rps12* intron 1	Aryamanesh et al., 2017; Lee et al., 2019
	ZmPPR4	At5g04810	Os04g58780	*rps12* intron 1	Schmitz-Linneweber et al, 2006; Lee et al., 2019
	PpPTSF1			$tRNA^{Ile}$ intron	Goto et al., 2016
	AtPBF2	At3g42630	Os08g40870	*ycf3* intron 1	Wang et al., 2020a
	AtSOT5	At1g30610	Os01g48380	*rpl2* intron, *trnK* intron	Huang et al., 2018
	OsPPR6		Os05g49920	*ycf3* introns	Tang et al., 2017
	OsSLC1	At5g42310	Os06g0710800	*rps16* intron	Lv et al., 2020
	AtEMB1270	AT3G18110	Os06g09880	*ycf3* intron 1, *clpP* intron 2*, *ndhA* intron	Zhang et al., 2021
	AtPDM3	AT5G39980	Os01g61630	*ndhB* intron, *trnA* intron, *clpP* intron1*	Zhang et al., 2017
	AtPDM4	At5g27270	Os06g02120	*ycf3* intron 1 and *clpP* intron 2*	Chen et al., 2020; Wang et al., 2020b
	AtACM1	AT3G18110	Os06g09880	*ndhA* intron, *ndhB* intron, *clpP* intron 2*, and *ycf3* intron 1	Wang et al., 2021a

Table 1. Continued

Type	Name	A.thaliana	O.Sativa	Affected introns	References
	AtECD2	AT5G50280	Os05g22870	*ndhA* intron, *rps12* intron 2, *ycf3* intron 1, and *clpP* intron 2*	Wang et al., 2021b
	OsWSL4	At1g09900	Os02g35750	*rps12* intron 2, *ndhA* intron, *atpF* intron, *rpl2* intron	Wang et al., 2017
	OsWSL5	At5g04810	Os04g0684500	*rpl2* intron, *rps12* intron 2	Liu et al., 2018
	ZmEMB-7L	At2g41720	Os07g11280	*rps12* intron 1, *rpl2* intron, *atpF* intron, *ndhA* intron	Yuan et al., 2019
	AtOTP70	At4g25270	Os03g63560	*rpoC1* intron*	Chateigner-Boutin et al., 2011
	OsWSL	At3G53700	Os01g37870	*rpl2* intron	Tan et al., 2014
	OsSLA4	AT5G03800	Os07g0172600	*petB* intron, *ndhA* intron, *atpF* intron, *rpl2* intron, *rpl16* intron, *trnG* intron, *rps12* intron 2	Wang et al, 2018
	OsPGL12		Os12g10184	*ndhA* intron	Chen et al., 2019
	AtPDM1	At4g18520	Os03g44210	*ndhA* intron, *trnK* intron	Zhang et al., 2015
	AtAEF1	AT3G22150	Os04g51350	*atpF* intron	Yap et al., 2015
	AtHCF152	At3G09650	Os12g0102600	*petB* intron	Meierhoff et al., 2003
	ZmPPR5	At4g39620	Os02g51480	*trnG* intron	Williams-Carrier et al., 2008
CRM proteins	ZmCRS1	At5g16180	Os08g27150	*atpF* intron	Till et al., 2001; Ostersetzer et al., 2005
		At4g29750	Os09g19850	*ndhA* intron, *petD* intron, *ndhB* intron, *ycf3* intron 1, *trnL* intron	Liu et al., 2016
		At3g18390	Os05g47850	unknown	

Table 1. Continued

Type	Name	A.thaliana	O.Sativa	Affected introns	References
	OsAL2	At4g29750	Os09g19850	*ndhA* intron, *petD* intron, *ndhB* intron, *ycf3* intron 1, *trnL* intron	Liu et al., 2016
	ZmCAF1	At2g20020	Os01g31110	*petD* intron, *trnG* intron, *rps16* intron, *rpl16* intron, *ndhA* intron, *ycf3* intron 1, *rpoC1* intron*, *ClpP* intron1*	Asakura and Barkan, 2006; Ostheimer et al., 2003
	ZmCAF2	At1g23400	Os01g21990	*petB* intron, *ndhB* intron, *ndhA* intron, *rps12* intron 1, *ycf3* intron 1	Ostheimer et al., 2003
	AtCFM2	At3g01370	Os04g39060	*trnL* intron, *ndhA* intron, *ycf3* intron 1, *clpP* intron 2*	Asakura and Barkan, 2007
	OsCAF1	AT2G20020	Os01g04959 00	*atpF* intron, *rpl2* intron, *rps12* intron, *ndhB* intron, *ndhA* intron, *ycf3* intron	Zhang et al., 2019
	OsCAF2	At1g23400	Os01g21990	*rpl2* intron, *rps12* intron, *atpF* intron, *ndhA* intron, *ndhB* intron, *ycf3* intron	Shen et al., 2020
	OsCFM2	AT3G01370	Os04g04648 00	*trnL* intron, *rps12* intron, *rpl2* intron, *ndhA* intron, *atpF* intron, *ycf3* intron 1	Zhang et al., 2020
	AtCFM3a	At3g23070	Os11g37990	*rps16* intron, *rpl16* intron, *ndhB* intron, *petB* intron, *petD* intron, *trnG* intron	Asakura et al., 2008
	AtCFM3b	At4g14510			Asakura et al., 2008
	OsCFM3	At3g23070; At4g14510	Os11g37990	*rps16* intron, *rpl16* intron, *petD* intron, *ndhB* intron	Asakura et al., 2008
	ZmCFM1	At3G18390	Os05g47850	*trnA* intron, *trnI* intron, *trnV* intron	Feiz et al., 2021

Table 1. Continued

Type	Name	A.thaliana	O.Sativa	Affected introns	References
peptidyl-tRNA hydrolase	ZmCRS2	At5G38290		*rps12* intron1, *ndhB* intron, *petB* intron, *ndhA* intron, *ycf3* intron1, *rpl16* intron, *rps16* intron, *petD* intron, *trnG* intron, *clpP* intron1* and *rpoC1* intron*	Jenkins et al., 1997; Jenkins et al., 2001
ribonuclease III proteins	ZmRNC1	At4g37510	Os01g59510	*rps12* intron 2, *atpF* intron, *trnK* intron, *trnV* intron, *trnI* intron, *trnA* intron, *petB* intron, *petD* intron, *ndhB* intron, *trnG* intron	Watkins et al., 2007
DUF860 or PORR	ZmWTF1	At4g01037	Os05g49610	*rps12* intron 2, *atpF* intron, *trnK* intron, *trnV* intron, *trnI* intron, *trnA* intron, *petB* intron, *petD* intron, *ndhB* intron, *trnG* intron	Kroeger et al., 2009
Whirly protein	ZmWHY1	At1g14410; At2g02740	Os06g05350	*atpF* intron	Prikryl et al., 2008
APO	AtAPO1	At1g64810	Os04g52520	*ycf3* intron 2, *petD* intron, and *clpP* intron 1*	Watkins et al., 2011
DEAD box RNA helicase	AtRH3	At5g26742	Os03g61220	*trnA* intron, *trnI* intron, *rpl2* intron, *rps12* intron, *trnL* intron, *trn*K intron, *atpF* intron	Asakura et al., 2012; Gu et al., 2014
mTERF protein	Zm-mTERF4	At4g02990	Os05g33500	*trnI* intron, *trnA* intron, *rpl2* intron	Hammani and Barkan, 2014
	At-mTERF2	At2g21710	Os07g04230	*ycf3* intron 1 and *rps12* intron 1	Lee et al., 2021

Asterisk (*) represents introns that are found in Arabidopsis thaliana but not in maize and rice. Orthologous genes in Oryza sativa or in Arabidopsis thaliana have been determined using Phytozome 13 (Phytozome BLAST Search (doe.gov)) or NCBI (National Center for Biotechnology Information (nih.gov)).
